# Simvastatin Efficiently Lowers Small LDL-IgG Immune Complex Levels: A Therapeutic Quality beyond the Lipid-Lowering Effect

**DOI:** 10.1371/journal.pone.0148210

**Published:** 2016-02-03

**Authors:** Gerd Hörl, Harald Froehlich, Ulrika Ferstl, Gerhard Ledinski, Josepha Binder, Gerhard Cvirn, Tatjana Stojakovic, Michael Trauner, Christoph Koidl, Erwin Tafeit, Karin Amrein, Hubert Scharnagl, Günther Jürgens, Seth Hallström

**Affiliations:** 1 Institute of Physiological Chemistry, Center of Physiological Medicine, Medical University of Graz, Graz, Austria; 2 Division of Angiology, Department of Internal Medicine, Medical University of Graz, Graz, Austria; 3 Division of Cardiology, Department of Internal Medicine, Medical University of Graz, Graz, Austria; 4 Clinical Institute of Medical and Chemical Laboratory Diagnostics, Medical University of Graz, Graz, Austria; 5 Division of Gastroenterology and Hepatology, Department of Medicine III, Medical University of Vienna, Vienna, Austria; 6 Institute of Hygiene, Medical University of Graz, Graz, Austria; 7 Division of Endocrinology and Metabolism, Department of Internal Medicine, Medical University of Graz, Graz, Austria; Showa University School of Pharmacy, JAPAN

## Abstract

We investigated a polyethylene glycol non-precipitable low-density lipoprotein (LDL) subfraction targeted by IgG and the influence of statin therapy on plasma levels of these small LDL-IgG-immune complexes (LDL-IgG-IC). LDL-subfractions were isolated from 6 atherosclerotic subjects and 3 healthy individuals utilizing iodixanol density gradient ultracentrifugation. Cholesterol, apoB and malondialdehyde (MDA) levels were determined in each fraction by enzymatic testing, dissociation-enhanced lanthanide fluorescence immunoassay and high-performance liquid chromatography, respectively. The levels of LDL-IgG-IC were quantified densitometrically following lipid electrophoresis, particle size distribution was assessed with dynamic light scattering and size exclusion chromatography. The influence of simvastatin (40 mg/day for three months) on small LDL-IgG-IC levels and their distribution among LDL-subfractions (salt gradient separation) were investigated in 11 patients with confirmed coronary artery disease (CAD). We demonstrate that the investigated LDL-IgG-IC are small particles present in atherosclerotic patients and healthy subjects. In vitro assembly of LDL-IgG-IC resulted in particle density shifts indicating a composition of one single molecule of IgG per LDL particle. Normalization on cholesterol levels revealed MDA values twice as high for LDL-subfractions rich in small LDL-IgG-IC if compared to dominant LDL-subfractions. Reactivity of affinity purified small LDL-IgG-IC to monoclonal antibody OB/04 indicates a high degree of modified apoB and oxidative modification. Simvastatin therapy studied in the CAD patients significantly lowered LDL levels and to an even higher extent, small LDL-IgG-IC levels without affecting their distribution. In conclusion simvastatin lowers levels of small LDL-IgG-IC more effectively than LDL-cholesterol and LDL-apoB levels in atherosclerotic patients. This antiatherogenic effect may additionally contribute to the known beneficial effects of this drug in the treatment of atherosclerosis.

## Introduction

Development and progression of atherosclerosis are associated with elevated levels of LDL and oxidized LDL (oxLDL) [1]. A hallmark of atherosclerosis is the uptake of modified forms of LDL via scavenger receptors leading to the transformation of macrophages and smooth muscle cells into foam cells [2]. LDL particles are modified in arterial intima and in the circulation by several mechanisms, such as glycation, lipolysis, aggregation and oxidation [3]. In addition to hypercholesterolemia, innate and adaptive immune mechanisms play a critical role in atherogenesis. Characteristics of autoimmune disease are present in atherosclerosis and vice versa accelerated atherogenesis is observed in autoimmune disease [4–8]. Autoimmune reactions targeting modified LDL particles are considered to contribute to atherogenesis as the resulting LDL-IgG-IC are effectively taken up by macrophages and other cell types via Fcγ-receptors [5, 6, 9, 10].

In autoimmune disease the removal of IC is dependent on size and structure of the complex. Very large and large IC are delivered mainly to the spleen and liver whereas small soluble IC persist in circulation and are likely to penetrate the endothelial barrier of blood vessels inducing atherogenic effects by triggering inflammatory processes [11]. LDL autoantibodies recognize epitopes formed by oxidation and glycation of apoB as well as modified phospholipids such as phosphorylcholine [5, 12–15]. However, most studies characterized circulating LDL-containing IC after polyethylene glycol (PEG) precipitation from sera. These PEG precipitable LDL-containing IC have been found in diabetic and atherosclerotic patients but also in healthy subjects [16–19].

To our knowledge only two studies report on the effect of statin treatment on PEG precipitable IC [20, 21]. They both show that statins lower PEG precipitable IC and lipids (LDL-cholesterol, apoB) to a similar extent. In addition, circulating LDL-IgG-IC have been quantified in plasma of patients with acute coronary syndromes by ELISA [22, 23]. At the best statin treatment of these patients resulted in a decrease in LDL-IgG-IC levels equal to the lipid lowering effect.

Another approach to characterize circulating LDL-IgG-IC from patients with coronary atherosclerosis was to primary isolate the IC from serum by affinity chromatography using anti-human IgG-agarose. The isolated IC were further purified by salt gradient ultracentrifugation. The authors suggest that multiple-modified desialylated LDL is the circulating autoantigen for anti-LDL autoantibodies [24].

Our novel approach in this study was to try to identify and characterize LDL-IgG-IC in LDL-subfractions after an initial purification step by ultracentrifugation. Ultracentrifugation separates LDL-IgG-IC from unbound proteins (free IgG) that would disturb subsequent analytical procedures. The use of single-step iodixanol gradient ultracentrifugation assures a very high level of preservation of particle integrity and enabled us to examine the presence of LDL-IgG-IC in supernatants from PEG precipitation experiments. In a second approach utilizing the classical two-step salt gradient ultracentrifugation we focused on the effect of statin therapy on the level and distribution of these specific LDL-IgG-IC in patients with CAD.

The following questions were addressed in the present study: (i) Is there a particular LDL-subfraction which is preferentially targeted by IgG? (ii) Is there a correlation between LDL-IgG-IC levels and the MDA content of a given LDL-subfraction (indicating oxidative modification)? (iii) Do these LDL-IgG-IC have a defined size and antigen/antibody ratio? (iv) Are these LDL-IgG-IC precipitable with PEG? (v) How does simvastatin therapy affect the level and distribution of these LDL-IgG-IC?

## Material and Methods

### Study subjects

The study was approved by the local ethics committee (Ethikkommission der Medizinischen Universität Graz). Approval number: 19–327 ex 07/08. The study subjects were enrolled at the Medical University of Graz (Department of Internal Medicine). The study was approved by the local ethics committee and written informed consent was obtained from all study subjects.

#### Group A (iodixanol gradient)

The study group included 3 female and 3 male patients (age range 60–92 years). The 6 patients suffered from peripheral artery occlusive disease (PAOD) with restenosis and were considered high-grade atherosclerotic. Blood collected from these patients and from 3 healthy controls was used for isolation of LDL-subfractions following single-step iodixanol gradient ultracentrifugation.

#### Group B (salt gradient)

This study group included 11 male statin-naive patients aged 35–75 years with confirmed CAD (assessed by coronary angiography). Subjects were treated with simvastatin (40 mg/day) for a period of three months. LDL-subfractions were isolated applying salt gradient ultracentrifugation.

### Materials

All materials were from Sigma-Aldrich; Austria, unless indicated otherwise.

#### Blood sampling

EDTA-plasma or serum was collected after an overnight fast. In group B blood was drawn at baseline and after 3 months of simvastatin therapy as described previously [25].

#### Isolation of lipoprotein fractions

**A: Iodixanol density gradient ultracentrifugation:** Lipoprotein subfractions were prepared from PAOD patients (group A) and healthy control subjects by self-generated iodixanol gradient single-step ultracentrifugation using a modification of the protocols of Davies et al. and Yee et al. [26, 27]. In brief 3.0 mL EDTA-plasma were mixed with 5.8 mL phosphate buffered saline (PBS; pH 7.4) and 3.2 mL iodixanol 60% (OptiPrep^®^ density gradient medium) and centrifuged in open-top polyclear tubes (Seton; Science Services GmbH; Munich, Germany) at 16°C and 36,000 rpm (222,000 g) for 67 h (swinging bucket rotor SW 41 Ti; Beckman Coulter GmbH). A total of 25 fractions (the bottom fraction was excluded) were isolated in steps of 3.0 or 1.0 mm by a piston gradient fractionator (Biocomp; Science Services GmbH; Munich, Germany). Lipoprotein fractions were analysed for density (DMA 58 density meter; Anton Paar GmbH, Graz, Austria), total cholesterol (“Cholesterin -CHOD-PAP reagent”; Greiner Diagnostic GmbH, Bahlingen, Germany), apoB, dissociation-enhanced lanthanide fluorescence immunoassay (DELFIA) and LDL-IgG-IC content (visualized by blots prepared after lipid electrophoresis).

**B: Salt density gradient ultracentrifugation:** Lipoprotein fractions (very low-density lipoprotein (VLDL), intermediate-density lipoprotein (IDL), LDL and high-density lipoprotein (HDL)) from CAD patients (group B) were prepared from 6 mL of plasma by preparative ultracentrifugation. Subsequently LDL (density: 1.019–1.065 g/mL) was further fractionated into six density subfractions by equilibrium density gradient centrifugation [25, 28]. Density ranges of the subfractions were: LDL-1, 1.019–1.031 g/mL; LDL-2, 1.031–1.034 g/mL; LDL-3, 1.034–1.037 g/mL; LDL-4, 1.037–1.040 g/mL; LDL-5, 1.040–1.044 g/mL; LDL-6, 1.044–1.065 g/mL. The preparation steps were performed in the presence of antioxidants (10 μmol/L trolox, 20 μmol/L 2,6-Di-tert-butyl-4-methylphenol, 100 μmol/L diethylenetriaminepentaacetic acid (DTPA)) and a protease inhibitor (12 μg/mL Pefabloc (Merck; VWR; Austria)). All lipoprotein fractions were dialysed in isotonic buffer (0.1 mol/L Tris-HCl, 0.15 mol/L NaCl, 100 μmol/L DTPA; pH 7.4) for 48 h (dialysis tubing MW cut-off 14,000 (Roth; Austria)). Lipids, apoB and total protein were measured in the lipoprotein fractions as described previously [25, 29].

#### Malondialdehyde analysis

MDA was determined according to a previously described high-performance liquid chromatography (HPLC) method after derivatization with 2,4-dinitrophenylhydrazine (DNPH) [30]. For alkaline hydrolysis of protein bound MDA 25 μL of 6 mol/L sodium hydroxide was added to 0.125 mL of each subfraction (1.5 mL tubes) and incubated at 60°C (Eppendorf heater) for 30 min. The hydrolyzed samples were deproteinized with 62.5 μL 35% (v/v) perchloric acid and after centrifugation (14,000 g; 2 min) 125 μL of the supernatant were mixed with 12.5 μL DNPH solution and incubated for 10 min. This reaction mixture, diluted derivatized standard solutions (0.625–10.00 nmol/mL) and reagent blanks were injected into the HPLC system (injection volume: 40 μL). The MDA standard was prepared as previously described (33). The DNPH derivates (hydrazones) were isocratically separated on a 5-μm ODS hypersil column (150x4.6 mm) guarded by a 5-μm ODS hypersil column (10x4.6 mm; Uniguard holder; Thermo Electron Corporation; Cheshire, UK) with a mobile phase consisting of a 0.2% (v/v) acetic acid solution (bidistilled water) containing 50% acetonitrile (v/v). The HPLC separations were performed with an L-2200 autosampler, L-2130 HTA pump and L-2450 diode array detector (all: VWR Hitachi; Vienna; Austria). Detector signals (absorbance at 310 nm) were recorded and program EZchrom Elite (VWR) was used for data acquisition and analysis.

#### Lipoprotein electrophoresis

Lipoprotein subfractions were separated on 1% agarose gels as described elsewhere [29]. Lipoproteins were electroblotted onto nitrocellulose membrane and blots were incubated with an antibody directed against human IgG (affinity purified polyclonal antibody: goat-anti-human IgG conjugated to horseradish peroxidase (HRP); Roth GmbH&Co.KG, Germany). The SuperSignal^®^ West Femto Max. Sensitivity Substrate (PIERCE, Fisher Scientific GmbH; Austria) was used for blot development. Detection and analysis of signals were performed with the ChemiDoc MP System and software Image Lab 4.0.1 build 6 (all: Bio-Rad Laboratories Ges.m.b.H.; Vienna; Austria) and TotalLab Array v2009 (TotalLab Array; Newcastle; UK). Furthermore, we performed electrophoresis of LDL-subfractions (#11, #12, and #13; isolated after ultracentrifugation in steps of 1.0 mm) rich in LDL-IgG-IC and the major LDL-subfraction (#7; fraction without detectable LDL-IgG-IC) in 3% polyacrylamide gels under conditions described elsewhere [31].

#### Dot-Blot Analysis of LDL-IgG-IC

A Bio-Dot^®^ microfiltration apparatus (Bio-Rad Laboratories Ges.m.b.H.; Vienna; Austria) was used to transfer lipoprotein subfractions onto nitrocellulose membranes. The samples were diluted in Tris-buffered saline (TBS; 20 mM Tris-HCl; 500 mM NaCl; pH 7.5) and 100 μL were applied in triplicates. The blots were processed according to the protocol of the electrophoresis experiments.

#### Determination of density/buoyancy of LDL-IgG-IC

In a first approach to assemble small LDL-IgG-IC in vitro we used the most prominent LDL-subfraction (150 μL of fraction #8 (cholesterol concentration: 380 mg/dL) derived from the iodixanol gradient centrifugation. This LDL-subfraction did not contain detectable LDL-IgG-IC. The incubation was carried out using a biotinylated IgG antibody targeting human apoB-100 (6.8 μg antibody diluted in 1.4 mL PBS, molar LDL to antibody ratio: ~10:1). Within 1 min the antibody solution was added dropwise to the LDL solution under gentle mixing. Further incubation was carried out at room temperature (RT) for 30 min on a roller mixer. Subsequently the whole mixture was applied to a second iodixanol gradient ultracentrifugation step. The harvested fractions were diluted 1:150 in 0.01 mol/L phosphate buffer, pH 7.4 and 100 μL were used to coat microtiter plates. After washing, blocking and incubation with Eu-labelled Streptavidin (DELFIA; PerkinElmer; Austria) fluorescence counts were recorded by a DELFIA Research Fluorometer as described previously [29]. Density and cholesterol content of subfractions were determined. The density difference of subfractions representing peak levels of cholesterol and LDL-IgG-IC was calculated.

In a second approach we used the LDL-subfraction that showed the highest levels of LDL-IgG-IC. Approximately 5% of this LDL-subfraction represented LDL-IgG-IC and about 95% was uncomplexed LDL. This LDL-subfraction was incubated with an HRP-antibody directed against the F(ab')_2_ fragment of human IgG in order to calculate the density shift resulting from binding of the antibody to the IgG of LDL-IgG-IC. Incubations were performed with different amounts of the HRP-antibody (#109-036-006, Peroxidase-AffiniPure F(ab')_2_ fragment goat anti-human IgG, F(ab')_2_ fragment specific; Jackson ImmunoResearch Europe Ltd). The HRP-antibody concentration was adjusted to achieve an antibody:LDL particle ratio of 1:100, 1:1,000 and 1:10,000; no antibody was added in the control. After a second ultracentrifugation separation run the collected fractions (1.0 mm steps) were analysed for HRP activity, cholesterol content and the amount of LDL-IgG-IC (dot-blot analysis). The density difference of subfractions representing peak levels of HRP activity and LDL-IgG-IC (control) was calculated.

#### PEG precipitation of LDL-IC

Precipitation of LDL-IC was performed by incubation of equal volumes of serum and PEG solution in accordance to frequently applied protocols [16, 17, 32, 33]. An equal volume of freshly prepared 7% (w/v) PEG 8000 (in borate buffered saline (BBS), pH 8.4, sterilized by filtration through a 0.22 μm filter) was added dropwise to serum in polystyrene tubes that were continuously and gently vortexed. After precipitation of IC (18 h at 4°C) the tubes were centrifuged at 2060 g for 20 min at 4°C. Pooled supernatants (n = 6) were used for preparation of LDL-subfractions (iodixanol gradient ultracentrifugation). After lipid electrophoresis and blotting LDL-subfractions were analysed for presence or absence of LDL-IgG-IC. Total cholesterol content of supernatant subfractions and PEG precipitates were determined enzymatically as described above.

#### Determination of LDL-IgG-IC, total-LDL-IgG-IC and apoB by DELFIA

DELFIA plates were coated for 12 h (4°C) with 1 μg anti-human IgG γ-chain antibody purified from rabbit antiserum (Behring/Siemens Healthcare Diagnostics GmbH; Germany) diluted in 200 μL 0.01 mol/L phosphate buffered saline (PBS; pH 7.4). The plates were rinsed three times with 500 μL wash buffer (0.01 mol/L phosphate buffer, 0.15 mol/L NaCl, 0.05% TWEEN^®^ 20, pH 7.4) using a microplate washer (Anthos Fluido, Biochrom; Austria). Subsequently 300 μL I-Block^™^ Blocking Reagent (0.2% in 0.01 mol/L phosphate buffer, 20 μmol/L EDTA, pH 7.4; Applied Biosystems; Austria) was applied for 1.5 h at RT. The dialysed LDL-subfractions were diluted according to the protein content to yield 2 μg LDL/200 μL (equivalent to ~400 ng apoB), transferred to the wells and incubated for 2 h at RT (horizontal shaker: 300 rpm). Subsequently, after washing 200 μL polyclonal biotinylated goat anti-human apoB-100 (Bio-Connect B.V, Huissen, The Netherlands) were added per well (dilution buffer: 0.01 mol/L phosphate buffer, 0.15 mol/L NaCl, 20 μmol/L EDTA, pH 7.4) and the dishes were incubated for 1 h at RT (shaking at 300 rpm). Finally after appropriate washing 200 μL DELFIA Eu-labelled Streptavidin (PerkinElmer; Austria; dilution 1:4,000 in 0.01 mol/L phosphate buffer, 20 μmol/L EDTA, pH 7.4) was added and incubated at RT for 1 h (shaking at 300 rpm). After 6 washing cycles the plates were incubated with 200 μL DELFIA enhancement solution (PerkinElmer; Austria) for 5 min on a shaker (Wallac Oy). Fluorescence counts were recorded by a DELFIA Research Fluorometer (Wallac Oy) as described previously [29]. In order to assess the absolute particle ratio of LDL-IgG-IC and LDL we determined LDL-IgG-IC and apoB levels using a very similar and comparable assay system (S1 Fig). ApoB content of LDL fractions was measured applying the same routine steps and detection antibodies as for the determination of LDL-IgG-IC levels. A goat anti-human apoB-100/48 antibody (Bio-Connect B.V, Huissen, The Netherlands) was used as capture antibody. A total LDL fraction (dilution range: 5–30 ng/mL) was used as apoB standard and the samples were diluted 1:10,000.

#### Determination of LDL modification

The monoclonal antibody OB/04 (IgG) was used to detect oxidatively modified apoB in LDL-IgG-IC according to a procedure described elsewhere [29]. An LDL- subfraction rich in LDL-IgG-IC was isolated from a healthy subject by iodixanol density gradient ultracentrifugation and incubated with magnetic beads (SiMAG—Protein A/G; Chemicell GmbH, Berlin, Germany) according to the manufacturer's protocol. The bound fraction was eluted with a glycine buffer (0.1 mol/L; 0.15 mol/L NaCl; pH 2.5) and neutralized with a Tris-HCl buffer (1 mol/L; pH 8.0). The bound fraction containing the LDL-IgG-IC and the unbound fraction (LDL-IgG-IC depleted fraction) were used to coat DELFIA plates at different dilutions (1:50, 1:150 and 1:200). As primary antibodies we used a rabbit IgG fraction directed against human apoB (CSL Behring; Marburg; Germany) for detection of total apoB (dilution 1:10,000) and the monoclonal mouse antibody OB/04 (dilution 1:200 (final conc. 5 μg/mL)) for detection of modified apoB epitopes. Europium labelled secondary antibodies were used to target primary antibodies (anti-rabbit IgG-Eu (R-4880) and anti-mouse-IgG-Eu (M-8770) at dilution 1:4,000 and 1:3,000, respectively). Washing, blocking, incubation and signal detection were performed as described for the other DELFIA procedures. The fluorescence counts acquired for modified apoB were normalized to fluorescence counts representing total apoB. The monoclonal antibody OB/04 reacts in solid-phase fluorescence immunoassays and western blot analysis with copper-oxidized LDL, LDL oxidized by a free radical-generating azo compound and copper-oxidized VLDL but not with native LDL, acetylated LDL, oxHDL3, azo-oxidized HDL3, or HDL3 modified with MDA. In competitive immunoassays with LDL modified by oxidized fatty acid-derived aldehydes OB/04 displays a weak affinity for LDL after modification with aldehydes (4-hydroxynonenal, 4-hydroxyhexenal, 4-hydroxyoctenal or hepta-2,4-dienal) except for MDA. LDL modified with arachidonic acid oxidation products (AAOPs) is also recognized by this antibody. Albumin modified either by the tested aldehydes or by AAOPs does not react with OB/04. OB/04 recognizes an epitope that is expressed only on apoB-containing lipoproteins upon oxidative modification [29]. As OB/04 selectively recognizes apoB structurally modified during oxidation of LDL we consider it as a class 2 antibody similar to the antibody 4E6 used in a commercially oxLDL test kit (Oxidized LDL ELISA, Mercodia AB; Uppsala; Sweden) [34].

#### Assessment of size of LDL-IgG-IC and uncomplexed LDL

High performance gel permeation chromatography with a TSK 5000 PW column (600 mm x 4 mm; Tosoh Bioscience LLC; Japan) was performed to elucidate the size distribution of an LDL-IgG-IC enriched subfraction according to the method described by Carroll and Rudel [35]. Fractions in the elution range of LDL were collected, transferred onto nitrocellulose (dot-blot) and analysed for presence of IgG. A flow rate 0.8 ml/min was applied and detection wavelength was 280 nm. The HPLC system used was the same as described for MDA analysis.

Dynamic light scattering was applied to assess particle sizes using a Zetasizer 3000 HS (Malvern Instruments, Herrenberg, Germany). An LDL-subfraction rich in LDL-IgG-IC (pooled LDL-IgG-IC peak fractions) was measured prior and after absorption of LDL-IgG-IC by beadBALL-Protein G (Chemicell GmbH, Berlin, Germany) according to the manufacturer's protocol. The complete removal of LDL-IgG-IC was confirmed by electrophoresis and immunodetection as described above.

#### Statistical analysis

ANOVA with post-hoc comparisons performed according to Scheffé was applied to assess significant differences of LDL-IgG-IC distribution (study group B) (SPSS Inc. Released 2009. PASW Statistics for Windows, Version 18.0. Chicago: SPSS Inc.). Shapiro-Wilks test was used to confirm normal distribution and Levene's test was applied to assess the equality of variances. For analysis of the effect of simvastatin therapy on cholesterol or apoB levels (one-sided, paired T test) and LDL-IgG immune complexes (two-sided test) the paired-samples T test was used. For estimation of differences of LDL-IgG-IC levels normalized to cholesterol or apoB levels a two-sided unpaired T test was used. A p-value < 0.05 was considered significant.

## Results

### Cholesterol, LDL-IgG-IC and MDA distributions among LDL-subfractions in PAOD patients (group A)

**Fig 1:**
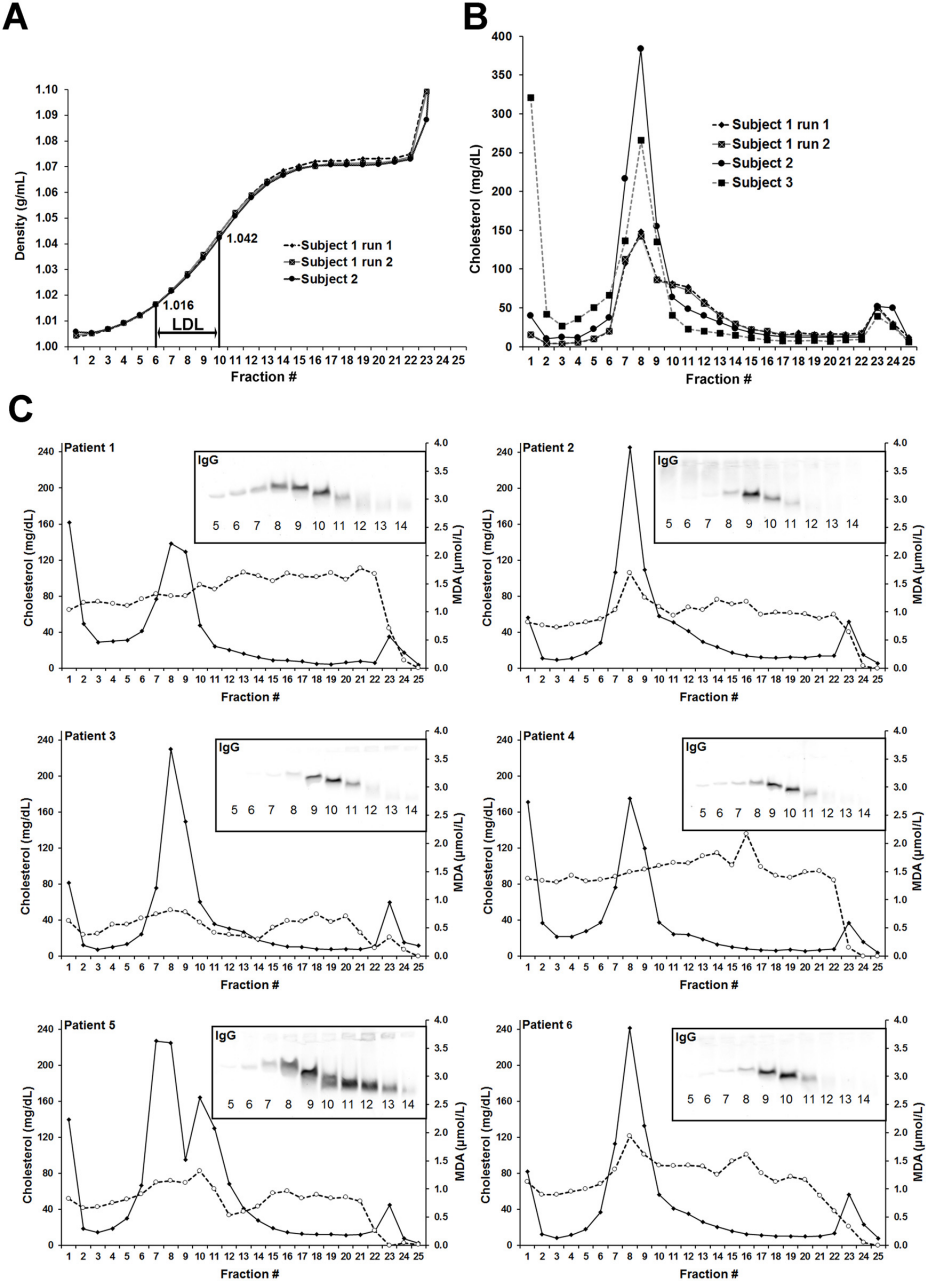
Identification of LDL-IgG-IC within the density range of LDL. Lipoproteins were separated by a self-generated iodixanol gradient single-step ultracentrifugation. Density of the obtained 25 subfractions and LDL region (**A**). Cholesterol levels of collected fractions from 3 healthy subjects (**B**). Distributions of cholesterol (solid lines), MDA (dotted lines) and LDL-IgG-IC detected by a specific anti-human-IgG antibody (insert at the top right) in patients with PAOD. MDA levels represent protein bound MDA (**C**).

The density gradient of the self-generated iodixanol single-step ultracentrifugation (range of LDL indicated) is shown in Fig 1A. The cholesterol distribution of lipoprotein subfractions from 3 apparently healthy subjects is presented in Fig 1B. One of these control subjects revealed signs of hypertriglyceridemia with fraction #1 cholesterol above 300 mg/dL and higher levels within the range of IDL. Lipoprotein (a) (Lp(a)) was present as a shoulder in fractions #10–12 (subject 1). Fig 1C illustrates the separation of lipoproteins from plasma of PAOD patients into 25 fractions of increasing density. The respective distributions of cholesterol and MDA as well as LDL-IgG-IC (inserts) are shown. Isolated LDL-IgG-IC fractions do not contain unbound IgG molecules (S2 and S3 Figs). Immunodetection of IgG revealed that unbound IgG was present in subfractions ≥ #16 (S2 Fig).

LDL-cholesterol concentration peaks in subfraction #8 (density: 1.028 g/mL). Interestingly, the peak of LDL-IgG-IC (LDL-subfraction #9 and #10) lags one or two subfractions behind the cholesterol peak fraction. The mean MDA concentration in the PAOD patients was 3.6 ± 1.2 nmol/mL. The mean MDA level of control subjects (n = 10) was markedly lower (1.86 ± 0.48 nmol/mL). In 3 out of 6 PAOD patients, MDA levels peaked, like LDL levels, in subfraction #8. Thus, the dominant LDL-subfraction apparently contains high or the highest (absolute) amounts of oxidatively modified LDL. Moreover, if normalized to cholesterol levels, the MDA concentrations of subfractions containing the majority of LDL-IgG-IC (combined subfractions #9 and #10) were 1.7 ± 0.3 fold higher compared to that of combined subfractions #7 and #8. In addition, modifications of LDL from LDL-IgG-IC were detected with an oxidation-specific antibody (see below).

### IgG binding increases the density of LDL

**Fig 2:**
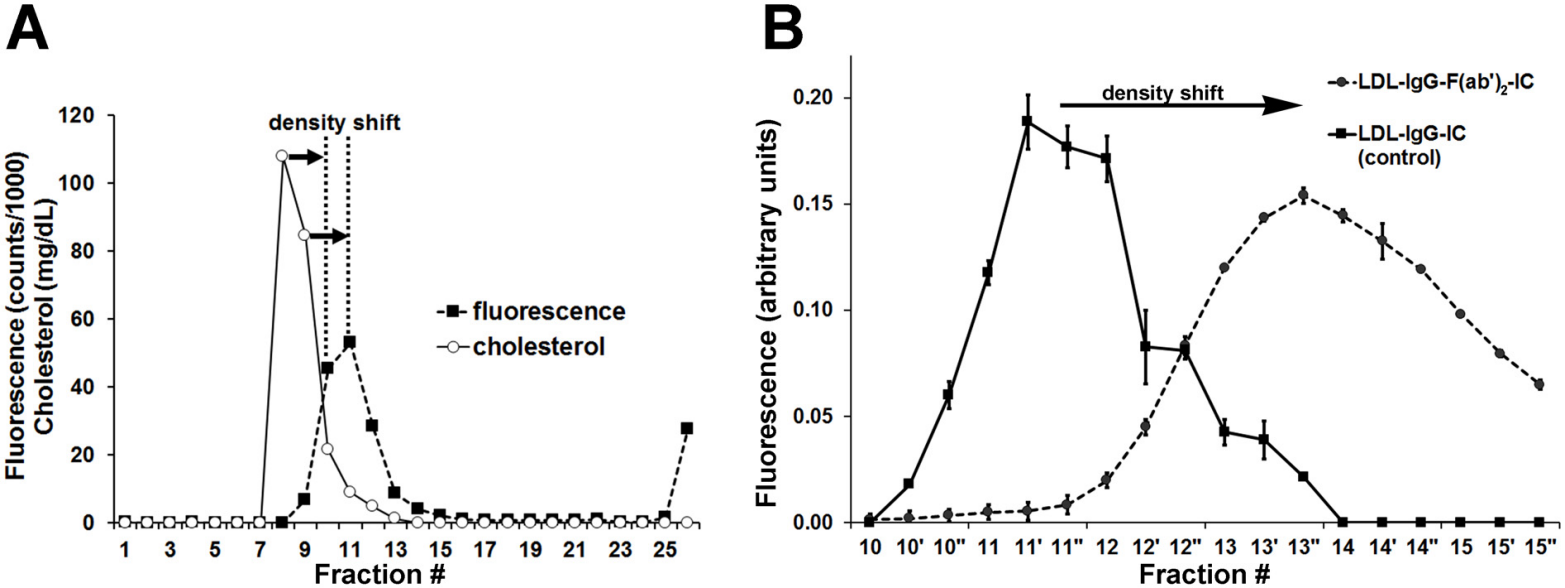
Identification of in vitro produced small LDL-IgG-IC by density shift. Small LDL-IgG-IC were produced by in vitro assembly of LDL-subfraction #8 and an anti-human apoB antibody (IgG). After self-generated iodixanol gradient single-step ultracentrifugation (fractionation step size: 3.0 mm) the cholesterol content was measured in the obtained subfractions and the presence of small LDL-IgG-IC was assessed by DELFIA (**A**). LDL-IgG-F(ab')_2_-IC consisting of an HRP-antibody fragment (targeting the F(ab)_2_ fragment of human IgG) and small LDL-IgG-IC were produced by incubation of an LDL-IgG-IC rich subfraction (#11) with the HRP-antibody fragment (antibody/LDL particle ratio of 1:100). After self-generated iodixanol gradient single-step ultracentrifugation fractionation was carried out with a step size of 1.0 mm. The density shift of LDL-IgG-IC (peak to peak difference) due to formation LDL-IgG-F(ab')_2_-IC is detected by measurement of HRP activity and immunodetection (dot-blot) of IgG in the control experiment (incubation without HRP-antibody fragment) (**B**).

Fig 2A illustrates a density shift of ~15 mg/mL (shift of approximately 2 fractions: ~6 mm) that is induced by binding of IgG. From the density gradient profile we calculated that a shift of 2 fractions (6 mm) corresponds to a density shift of 15.6 mg/mL. An increase in protein percentage induced by one single IgG perfectly explains the size of the observed density shift. Our assumption that exactly one single IgG molecule is present in the prepared LDL-IgG-IC is additionally confirmed by experiments showing that binding of an HRP-F(ab')_2_-antibody (MW: 190 kDa) targeting IgG of the small LDL-IgG-IC resulted in formation of LDL-IgG-F(ab')_2_-IC and a similar shift of ~6–7 mm in the centrifugation tube (fractionation step size: 1.0 mm), which corresponds to a similar density shift of ~15 mg/mL (Fig 2B).

### Identification of LDL-IgG-IC as small LDL-IgG-IC

**Fig 3:**
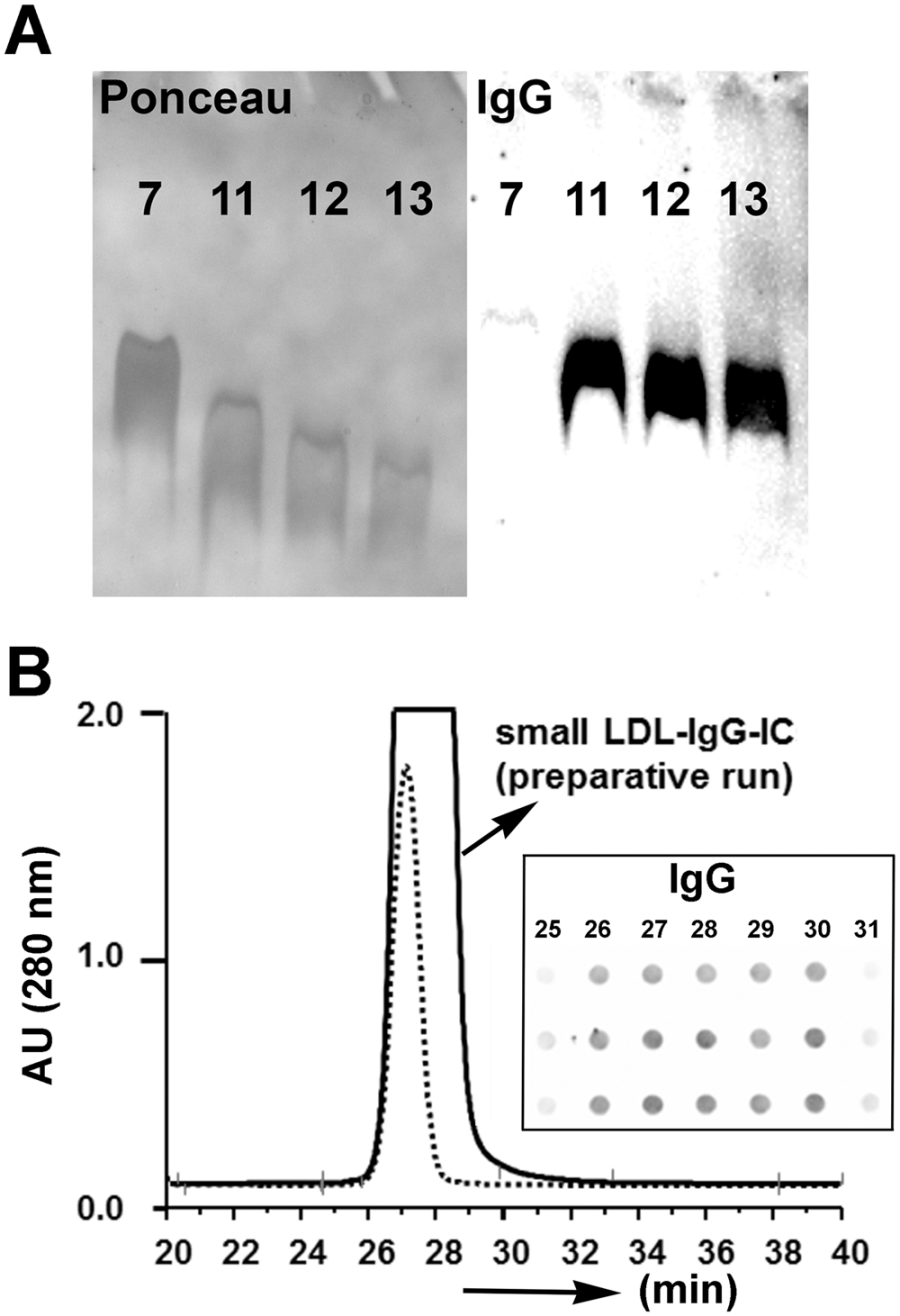
Estimation of LDL-IgG-IC particle size. Protein staining (left panel; Ponceau staining) and IgG immunodetection (right panel) of LDL-subfractions after electrophoresis in 3% polyacrylamide slab gel (migration is possible for particles < 35 nm). The small LDL-IgG-IC particles of subfractions #11, #12 and #13 (IgG) show a similar mobility like the major LDL-subfraction #7 (protein stain) (**A**). High performance gel permeation chromatography (TSK 5000 PW column; 600 mm x 4 mm) of a small LDL-IgG-IC enriched LDL-subfraction. Fractions of 1 min in the elution region of LDL were collected (solid line: preparative isolation; sample = 80 μL of LDL-IgG-IC enriched subfraction). The dotted line represents an analytical run sample = 20 μL of LDL-IgG-IC enriched subfraction). The distribution of LDL-IgG-IC (preparative run; fractions of min 25–31) in the eluted fractions was visualized after dot-blot analysis (immunodetection of IgG) (insert) (**B**).

In PAOD patients (Fig 1C) we observed a density difference between the peak of LDL-cholesterol (subfraction #8) and peak of LDL-IgG-IC (subfractions #9 and 10, inserts) of approximately 3–6 mm (~1–2 fractions). Electrophoresis experiments using 1% agarose and 3% polyacrylamide slab gels (Figs 1C and 3A) showed an electrophoretic behavior of LDL-IgG-IC similar to uncomplexed LDL particles indicating the absence of dimeric or multimeric structures. Data from gel permeation chromatography (Fig 3B) and dynamic light scattering (DLS) analysis indicate the small size and monomeric structure of LDL-IgG-IC. We, therefore, assume that a modified LDL particle is targeted by one single IgG molecule (see also: in vitro experiments).

### Removal of small LDL-IgG-IC does not change particle size distribution

DLS experiments revealed identical particle size distributions for the small LDL-IgG-IC enriched fraction (intensity averaged diameter 22.4 ± 6.7 nm) and the identical fraction after removal of small LDL-IgG-IC (22.3 ± 6.2 nm). Approximately 5% of LDL represent small LDL-IgG-IC (enrichment factor ~5) in these fractions. These results indicate the absence of large (multimeric) LDL-IgG-IC.

### Gel electrophoresis of small LDL-IgG-IC

Agarose and polyacrylamide gel electrophoresis displayed migration of LDL-IgG-IC as distinct bands indicating a narrow particle size range. Using 3% polyacrylamide slab gels LDL-IgG-IC present in #11, #12, #13 and #7 (LDL peak fraction) showed a very similar electrophoretic mobility (indicating similar particle size) (Fig 3A).

### High performance gel-filtration chromatography of small LDL-IgG-IC

During size exclusion chromatography of an LDL-IgG-IC enriched subfraction (~95% LDL; ~5% LDL-IgG-IC) LDL and LDL-IgG-IC eluted from the column with a very similar distribution profile (Fig 3B). Dot blot analysis of the eluted fractions showed that LDL-IgG-IC are present in all fractions of the collected peak fractions. Therefore, we suggest a similar size of LDL and LDL-IgG-IC in this subfraction and the absence of large multimeric complexes.

### LDL of small LDL-IgG-IC is oxidatively modified

Purified small LDL-IgG-IC (retained by protein A/G beads) and residual LDL (small LDL-IgG-IC depleted) were prepared from an LDL-subfraction rich in small LDL-IgG-IC. We compared the levels of oxidatively modified LDL by means of the monoclonal antibody OB/04 (directed against oxidation-specific epitopes) in these fractions. The levels of oxidatively modified LDL were increased ~70-fold in small LDL-IgG-IC (normalized to apoB levels) compared to unbound LDL (S4 Fig). We therefore conclude that oxidatively modified LDL is a preferred target of IgG.

### PEG precipitation of small LDL-IgG-IC

**Fig 4:**
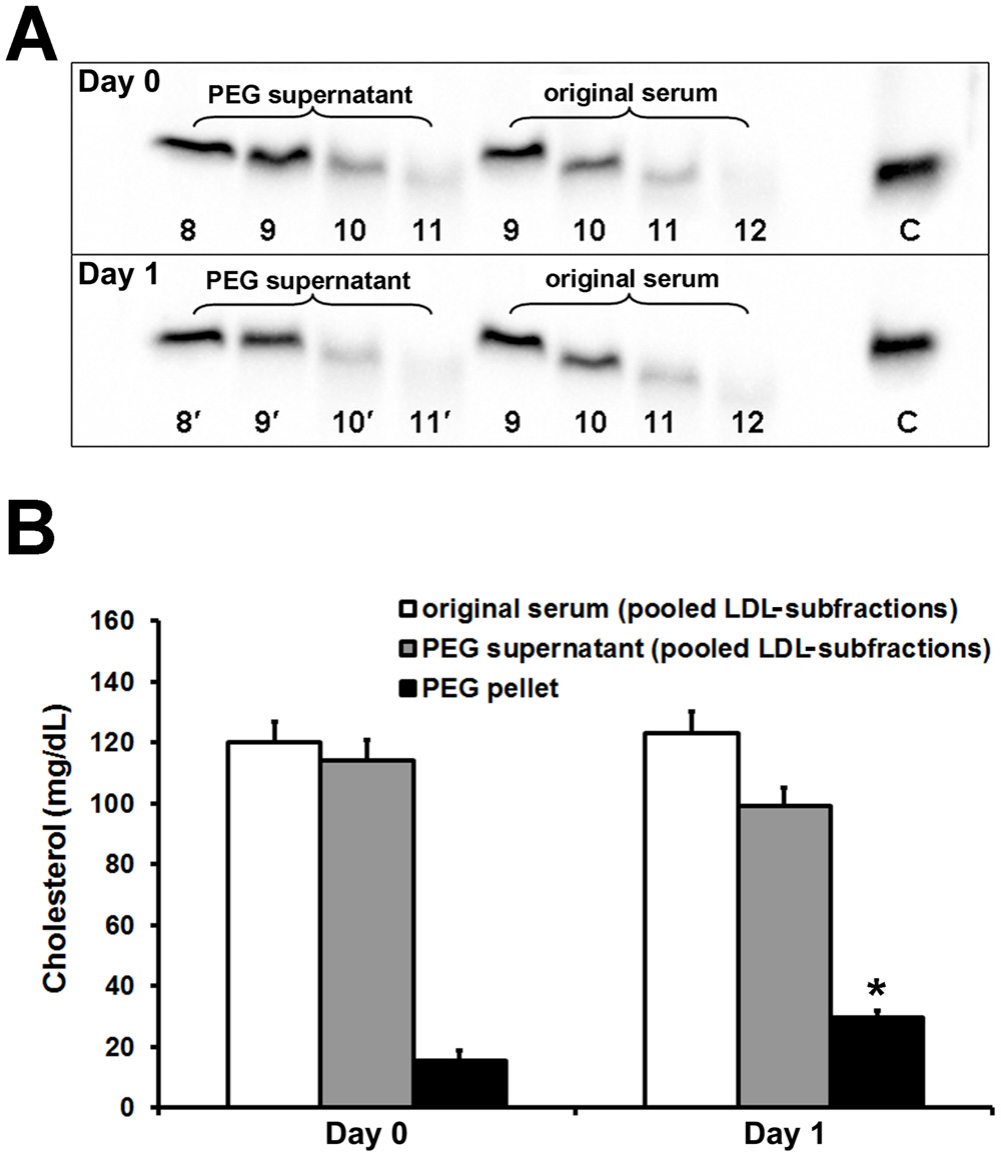
Small LDL-IgG-IC are not precipitable with PEG. Lipid electrophoresis of LDL-subfractions prepared (iodixanol gradient ultracentrifugation) from original serum and PEG supernatants after PEG 8000 precipitation (fractionation step size: 3.0 mm). Bands represent small LDL-IgG-IC present in the indicated LDL-subfractions visualized by immunodetection of IgG. **Day 0 (fresh serum):** LDL-subfractions #8–11 (from PEG supernatant) and LDL-subfractions #9–12 (original serum diluted with borate buffered saline to the same final volume). Band C: undiluted control (LDL-IgG-IC rich) subfraction isolated earlier from the same donor. **Day 1:** Repetition of the experiment with serum stored for 24 h at 4°C. The numbering of bands (PEG supernatant and original serum) is slightly different due to the density contribution of PEG. (**A**). Cholesterol amounts of combined LDL-subfractions (iodixanol ultracentrifugation) after PEG precipitation. Bars represent cholesterol concentrations (transformed to serum concentrations) of original serum (cholesterol of combined LDL-subfractions without PEG precipitation), PEG supernatant (cholesterol of combined LDL-subfractions after PEG precipitation) and PEG pellet (cholesterol content of precipitate) on day 0 (fresh serum) and day 1 (stored serum). *p < 0.001; increase of pellet cholesterol vs. day 0 (**B**).

PEG 8000 did not precipitate small LDL-IgG-IC from serum samples. Small LDL-IgG-IC are still present in the respective supernatants (Fig 4A). However, PEG reproducibly precipitated large amounts of LDL (also from cholesterol peak fractions that do not contain small LDL-IgG-IC (data not shown)) (Fig 4B). Electrophoresis of solubilized precipitates and immunoblotting revealed the presence of large amounts of IgG with electrophoretic mobility similar to unbound IgG (S3B Fig). Furthermore, an additional 24 h storage of serum at 4°C led to a decrease of cholesterol in the supernatant and a concomitant increase of PEG precipitable cholesterol (day 0 vs. day 1; p < 0.01; Fig 4B).

### Simvastatin effects on cholesterol, apoB and LDL-IgG-IC levels in CAD patients

Agarose gel electrophoresis of the 6 isolated subfractions and immunodetection of LDL-IgG-IC showed an electrophoretic mobility similar to subfractions derived from the iodixanol gradient ultracentrifugation. Determination of small LDL-IgG-IC levels with DELFIA (linearity and imprecision profiles of the LDL-IgG-IC DELFIA are shown in S1C Fig) revealed a distinct distribution pattern as assessed by ANOVA. The levels of small LDL-IgG-IC were significantly higher in the more dense subfractions (S5 Fig). Three out of 11 CAD patients did not respond to simvastatin treatment showing no reduction of plasma cholesterol. These non-responders were excluded from further evaluations. There was a significant reduction in LDL-cholesterol and LDL-apoB with an effect size of 37.4 ± 20.0% (one-sided, paired T test at p < 0.0005) and 34.1 ± 28.0% (one-sided, paired T test at p < 0.005), respectively.

**Fig 5:**
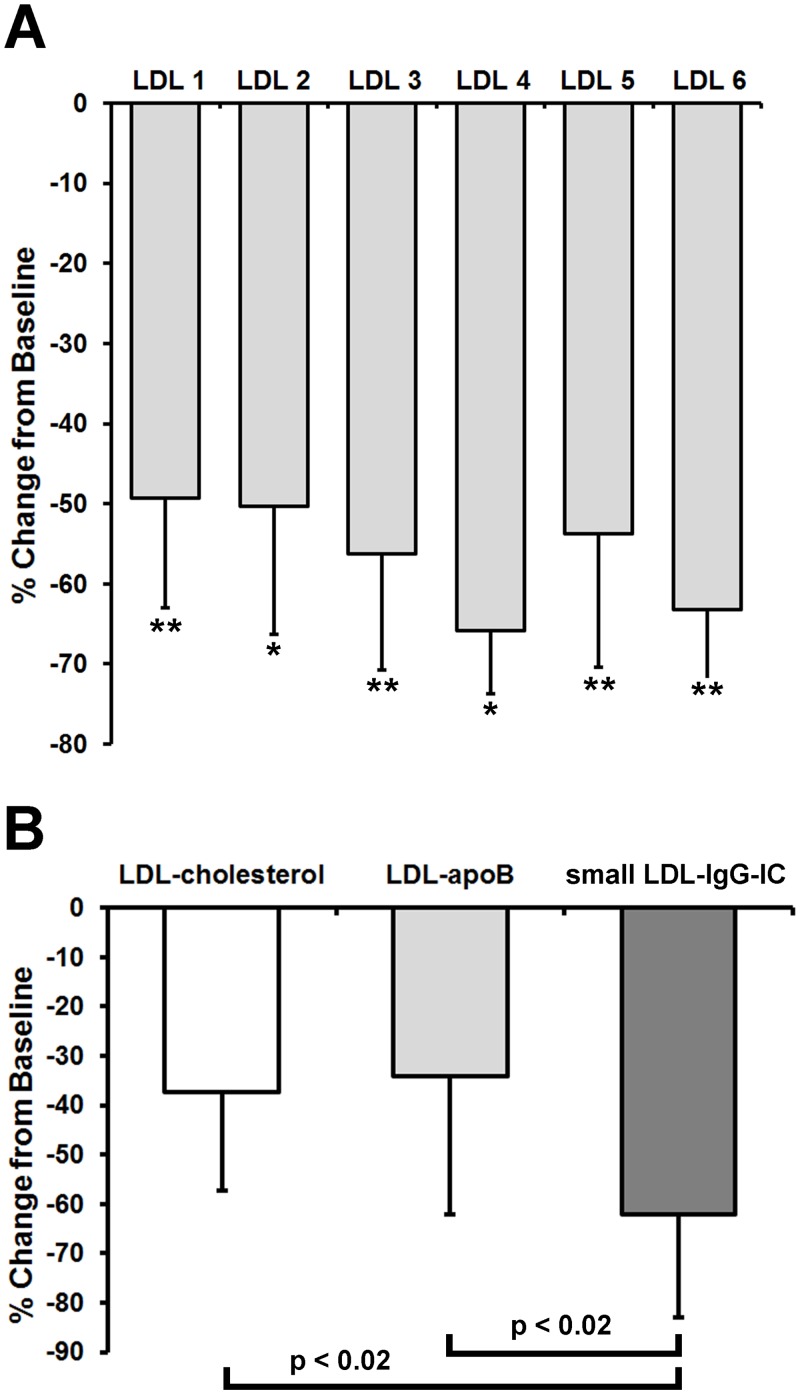
Simvastatin lowers small LDL-IgG-IC levels more effectively than cholesterol and apoB in patients with CAD. The reduction of total small LDL-IgG-IC levels is presented as percentage change from baseline for the 6 individual LDL-subfractions. Total amounts of small LDL-IgG-IC per fraction were calculated by conversion of the LDL-IgG-IC DELFIA counts. For each subject and each LDL fraction the baseline value and the value after statin therapy were used to calculate the difference as a percentage (post-statin minus pre-statin). Each bar represents the mean difference of total small LDL-IgG-IC per fraction on a percentage basis (*p < 0.05; **p < 0.01). (**A**). Comparison of the reduction of LDL-cholesterol, LDL-apoB levels and total small LDL-IgG-IC (average of reduction of LDL-subfractions) expressed as percentage change from baseline. Bars for LDL-cholesterol and LDL-apoB represent the percentage differences (post-statin minus pre-statin) determined in the entire LDL fractions. The reduction of the total amount of small LDL-IgG-IC of LDL is presented as average of the percentage differences calculated for LDL-subfractions 1–6 (shown in Fig 5A). Data represent means ± SD (**B**).

The reduction of total small LDL-IgG-IC levels expressed as percentage change from baseline for the 6 individual LDL-subfractions is shown in Fig 5A. Simvastatin treatment lowered small LDL-IgG-IC at least by ~50% in each LDL-subfraction (with a mean of 62.7 ± 11.9%). A comparison between the reduction of LDL-cholesterol, LDL-apoB levels and total small LDL-IgG-IC (average of reduction of LDL-subfractions) expressed as percentage change from baseline (Fig 5B) shows that the statin-induced decrease in small LDL-IgG-IC levels is significantly higher (approximately two-fold) when compared to LDL-cholesterol and LDL-apoB levels. The distribution pattern of small LDL-IgG-IC remained unchanged upon treatment with simvastatin indicating uniformity of the statin effect (S5A Fig). An averaged apoB DELFIA calibration curve was used to convert fluorescence counts from the LDL-IgG-IC DELFIA into apoB mass. Actual concentrations of apoB mass and particle ratio (IC-apoB as percentage of total apoB) of small LDL-IgG-IC are shown as part of S5C Fig.

### Percentage of small LDL-IgG-IC

Small LDL-IgG-IC and apoB DELFIA data were used to assess the percentage of small LDL-IgG-IC including all subjects evaluated so far. For combined LDL-subfractions (total LDL) we calculated that ~0.1–1% (combined LDL-subfractions) represent small LDL-IgG-IC. Due to the very work-intensive analytical process and strong individual variations of small LDL-IgG-IC levels it was impossible to work out significant differences between study groups.

## Discussion

The assessment of autoimmunogenicity of lipoproteins is complex and multi-faceted. However, it is generally accepted that LDL-IgG-IC bear atherogenic potential as the modification of LDL and the presence of the IgG-Fc region are both considered as driving factors behind foam cell formation [5, 6, 9, 10]. In this study we have characterized LDL-IgG-IC that were not PEG precipitable. Our approach to investigate LDL-IgG-IC included an initial density gradient ultracentrifugation step separating LDL from unbound IgG. Machine-aided fractionation of lipoprotein enabled us to accurately detect small density shifts and differences between lipoprotein fractions and to investigate the distribution and composition of LDL-IgG-IC regarding their antigen/antibody (LDL/IgG) ratio which is considered a determinant factor regarding clearance and atherogenic potential of LDL-IgG-IC.

LDL-IgG-IC particle integrity (presence of both apoB and IgG) was verified by DELFIA and immunodetection following lipid electrophoresis. The distribution patterns of LDL-IgG-IC and cholesterol are very similar in shape but show an offset of ~2 fractions. A similar offset was described for affinity purified LDL-IgG-IC. Tertov et al. attributed the observed density shift to the binding of IgG and/or desialylation of LDL [24]. To our knowledge, only one single study provides an LDL/IgG ratio of affinity purified LDL-IgG-IC from patients with ischaemic heart disease with a value of 2:1 indicating a dimeric structure. However, no experimental evidence was provided in this study [36]. In our setting the antigen/antibody (LDL/IgG) ratio was assessed by analysing the density shift caused by binding of one single IgG particle to LDL and to LDL-IgG-IC. Direct methods were not applicable as the affinity purified LDL-IgG-IC tend to decompose when stripped from the affinity column. Furthermore, appropriate reference molecules to determine absolute particle concentrations of IgG and apoB as integral part of LDL-IgG-IC are unavailable. The in vitro formation of LDL-IgG-IC and LDL-IgG-F(ab')_2_-IC results in density shifts that perfectly fit to an LDL/IgG ratio of 1:1. Upon binding of one single IgG the protein mass percentage of LDL (dominant fraction: 22.2%) is increased by 4.4%. Based on experimental and emulsion particle model data particle density would be raised by 13.2, 15.0 or 16.1 mg/mL [37, 38]. These density shifts correspond to distance shifts of 5.1, 5.8 and 6.2 mm in the iodixanol gradient setting which is in good agreement with our experimental data.

Based on our results and published experimental data we conclude that the investigated subset of LDL-IgG-IC most likely originates from the dominant LDL-subfractions. A priori we expected a higher IC formation tendency of more dense LDL-subfractions as these fractions are considered to be highly atherogenic [39–43]. Our results indicate that the investigated LDL-IgG-IC are small, monomeric, non-aggregated and non-latticed particles. The applied DELFIA system enabled us to determine the LDL-IgG-IC/LDL particle ratio. In the studied subjects ~0.1–1% of total LDL represented small LDL-IgG-IC. Itabe et al. calculated that ~0.01% of total LDL represent oxLDL in healthy subjects [44] and even if compared to elevated levels of oxLDL in atherosclerotic subjects the levels of found small LDL-IgG-IC are even higher and must therefore be considered as atherogenic factor [45]. However, we observed large individual differences in small LDL-IgG-IC levels and the study group sizes were too small to assess further correlations. After affinity purification of LDL-IgG-IC a high degree of modified apoB was detected. These epitopes of LDL-IgG-IC were detected with the monoclonal antibody OB/04 and are probably related to oxidative modifications. The OB/04 antibody reacts with copper-oxidized LDL but not with MDA-LDL. Upon immunohistochemical staining the same antibody detected macrophage-derived foam cells in human atherosclerotic plaques [29]. Furthermore, the determination of MDA in our subfractions revealed that the absolute MDA concentration did not show strong variations. Peak fractions of small LDL-IgG-IC (#9/#10) show approximately twofold higher MDA levels than LDL peak fractions (#7/#8) when normalized on cholesterol content. We therefore assume that MDA may not appear as the primary autoimmunogenic factor in small LDL-IgG-IC formation. Considering MDA as major immunogenic epitope of LDL-IgG-IC one would expect fractions rich in LDL-IgG-IC to show elevated MDA levels. However, the observation that MDA is widely distributed without peak levels in fractions rich in LDL-IgG-IC may indicate that MDA is probably not the major immunogenic epitope. The presence of elevated modified apoB epitopes recognized by the OB/04 antibody indicates the presence of immunogenic epitopes different from MDA. Hunt et al. reported that over 90% of modified LDL in circulation is associated to specific antibodies circulating as part of immune complexes [46]. As consequence, small LDL-IgG-IC can contain all possible types of modified LDL including oxidized phospholipids, advanced glycosylation end-products and MDA-LDL [3, 5, 12–19, 22–24, 46–48]. We assume that various modifications of LDL may result in structural changes of apoB recognized by IgG. Tertov et al. investigated LDL-IgG-IC isolated by ultracentrifugation (within the density range of LDL) providing evidence that desialylated LDL predominately interacts with antibodies forming LDL-IgG-IC and suggested that multiple-modified desialylated LDL is the circulating autoantigen for anti-LDL autoantibodies [24]. Desialylated LDL is considered to be identical with electronegative LDL recently reported to be able to induce the release of interleukin (IL)-1β and to activate the NLRP3 inflammasome in endothelial cells and leukocytes [49, 50]. We consider that LDL present in small LDL-IgG-IC and electronegative/desialylated LDL are probably related or identical particles.

The important question which modification of LDL is primarily responsible for IgG targeting and formation of IgG-IC is still unanswered. The investigated small LDL-IgG-IC contain increased amounts of structurally modified apoB due to chemical modification of the protein and/or the lipid components of LDL. However, IgG is detectable within the density range of VLDL, IDL, LDL and Lp(a) probably indicative of an immunogenic role of modified apoB. We assume that modified apoB is a recognized pathogen able to initiate an adaptive immune response. Due to its size and plasma half-life we consider oxLDL as a likely candidate to act as a trigger particle of the immune response against modified apoB. However, the IgG antibodies may result from a polyclonal response targeting a variety of LDL associated epitopes.

Several native and MDA-modified peptide sequences in apoB have been identified that were recognized by autoantibodies in human plasma. The immune responses against native apoB may be of equal importance as the presence of apoB reactive CD4^+^ T cells implies that regulatory T cells (Tregs) exist to control these apoB-reactive T cells. When the balance between apoB-reactive effector T cells and Tregs is shifted in favour of the effector T cells, a local loss of tolerance against LDL in the plaque could aggravate atherosclerosis [51].

Migration of LDL-IgG-IC in 1% agarose and 3% polyacrylamide slab gels showed an electrophoretic mobility similar to uncomplexed LDL particles indicating the absence of multimeric structures. Gel permeation chromatography and DLS analysis confirmed our hypothesis about the small size and monomeric (non-latticed) structure of LDL-IgG-IC. As determined by Gutierrez at al. the intensity-averaged diameter of a dimeric LDL-IgG-IC is ~34 nm and ~23 nm for monomeric LDL [52]. Particle sizes of a small LDL-IgG-IC enriched fraction were in the range of monomeric LDL. After removal of LDL-IgG-IC from this fraction the size distribution remains unchanged. Tertov et al. investigated affinity purified LDL-IgG-IC from serum of healthy and atherosclerotic subjects and showed that the electrophoretic mobility was increased by 39% (relative to sialylated LDL) with an estimated particle size of 19.5 nm for LDL-IgG-IC and 24.0 nm for LDL [24]. The particle size is an important factor defining the probability for small LDL-IgG-IC to enter compromised endothelial barriers and to escape clearance pathways specific for large-sized (latticed) IC [11, 53].

In our PEG experiments the precipitated cholesterol amounts were in good agreement with published values [16–19]. However, using PEG 8000 small LDL-IgG-IC were not precipitable. Furthermore, we observed a significant increase in pellet cholesterol after storage of serum at 4°C and concomitantly, a storage-time-dependent decrease in cholesterol of LDL-subfractions isolated from PEG supernatants. Apart of minimal aggregation, storage of serum samples at -80°C did not affect PEG precipitation (data not shown). In order to preserve stability and integrity of LDL during applied procedures and upon storage we use freshly drawn blood samples for preparation of subfractions.

One major goal of this study was to examine if small LDL-IgG-IC present in LDL-subfractions would respond to statin treatment. In our setting simvastatin was highly effective in lowering small LDL-IgG-IC without affecting their distribution. Total LDL-IgG-IC levels were reduced by 62.7% whereas the reduction of LDL-cholesterol and LDL-apoB was 29.7% and 26.0%, respectively. So far it has not been shown that statin treatment decreases LDL-IgG-IC to a higher extent than LDL [20, 22, 23]. A number of studies have suggested that statins have pleiotropic effects on immune response [54, 55]. LDL-IgG-IC may trigger a variety of effects as Fcγ-receptors are expressed by all kinds of blood cells except erythrocytes. It has been demonstrated that oxLDL-IgG-IC promote survival of monocytes by cross-linking Fcγ-receptor I an effect that may play a role in the accumulation of macrophages in human atherosclerotic lesions. OxLDL-IgG-IC activate complement and induce cytokine production by monomac 6 cells and human macrophages [56]. Furthermore, it also had been shown that not only modified apoB but also non-modified apoB can be targeted by the immune system [57, 58]. However, several studies demonstrate enhanced foam cell formation upon LDL-IgG-IC uptake by macrophages [5, 6, 9, 10] and hence, it appears plausible to suggest a similar role for small LDL-IgG-IC particles. The role of immune responses against modified self-antigens as oxLDL in atherosclerosis has been the focus of several studies during the last decades. Previous reports studying the associations between autoantibodies to oxLDL and cardiovascular disease have provided inconsistent results probably related to experimental difficulties as oxLDL is poorly defined as antigen and neo-eptiopes are continually formed and degraded during the oxidation process.

Antibodies of IgM subclass to phosphorylcholine and oxLDL are protective factors for atherosclerosis in patients with hypertension [14]. A clinical study including 508 men and 514 women demonstrated an inverse relation between anti-oxidized LDL IgM and carotid artery atherosclerosis [59]. Ravandi et al. investigated 748 cases and 1723 controls and observed an inverse association between levels of both IgM MDA-LDL and IgM IC and risk of CAD events in the subgroups with high levels of oxPL/apoB and Lp(a). These data suggest that levels of IgM MDA-LDL and IgM IC may modulate the proatherogenic effects associated with markers of oxidation such as oxPL/apoB and Lp(a) [60]. In a cohort of women with carotid plaques high levels of IgM against MDA-p210 were associated with less severe carotid disease [61]. The protective function of IgM is also supported by the development of accelerated atherosclerosis in IgM deficient mice [62].

Kobayashi et al. reported that IgM anti-oxLDL antibodies recognize the oxidized lipid moiety and IgG anticardiolipin antibodies recognize β2-glycoprotein I (β2GPI) complexed with oxLDL. Both types of antibodies bind to oxLDL/β2GPI complexes, but their roles on atherogenesis are opposite. The anti-oxLDL antibodies are anti-atherogenic and anticardiolipin antibodies in antiphospholipid syndrome (APS) are atherogenic. The authors assume that IgG anti-β2GPI antibodies contribute to lipid metabolism (housekeeping of oxLDL by macrophages) and that IgM anti-oxLDL antibodies are antiatherogenic. The oxLDL/β2GPI immune complexes may be internalized via FcγRI on macrophages [63].

It has been shown that oxLDL forms a stable and non-dissociable complex with β2GPI and that IgG anti-β2GPI autoantibodies are able to recognize this complex, thus facilitating macrophage-derived foam cell formation in patients with APS. However, the immunopathological mechanisms of oxLDL/β2GPI complexes in promoting foam cell formation are not fully understood. Zhang et al. demonstrated that toll-like receptor 4 plays an important role in the process of oxLDL/β2GPI/anti-β2GPI complex-induced transformation of macrophages to foam cells, which may accelerate the development of atherosclerosis in the setting of APS. It was also shown that β2GPI alone functions as an antiatherogenic protein by preventing the foam cell formation induced by oxLDL [64].

High levels of oxLDL/β2GPI complexes and anti-complex IgG as well as IgM have been reported in systemic lupus erythematosus (SLE) [65]. The titers of oxLDL/β2GPI were significantly higher in patients with renal involvement and previous thromboembolic episodes and were correlated with the number of risk factors for atherosclerosis, whereas they were significantly lower in patients with neurological involvement. Both IgG and IgM anti-complex antibodies were associated with antiphospholipid (APL). The oxLDL/β2GPI complex as well as antibodies against the complex are prevalent in SLE where they seem to be involved in organ damage.

Nowak et al. reported elevated serum concentration of IgG anti-oxLDL-β2GPI antibodies and IgM anti-oxLDL-β2GPI antibodies in the SLE group compared to the controls. There was a statistically significant positive correlation between LDL concentration and anti-oxLDL antibody concentration in the SLE group [66].

According to Hansson and Hermansson antigen-presenting cells (APC) take up native and modified LDL by LDL receptors and scavenger receptors, respectively. Uptake of IgG-IC could also be mediated by Fcγ-receptors. Presentation of peptides derived from apoB are recognized by CD4^+^ T cells and the ensuing T cell activation leads to cytokine secretion that can promote macrophage activation and inflammation. Once T cells are activated toward native apoB epitopes, they may support B cells recognizing native LDL, apoB, or lipids such as phosphocholine (PC), but also oxidatively modified epitopes such as MDA-apoB or oxPC [67].

Unexpectedly, Li et al. demonstrated that anti-oxLDL IgG immune therapy is capable of modulating macrophage pro-inflammatory activity through delivery of dominant inhibitory FcγR-signaling in vitro, and that it reduces inflammation and improves insulin sensitivity. The inhibitory effect is mediated through LDL-IgG-IC formation and is dependent on the antibody Fc fragment and FcγRII on responsive cells to transduce inhibitory intracellular signaling [68].

There exists strong evidence that FcγR play an important role regarding atherogenic potential of immune complexes. FcγR bind IgG monomers or IgG-IC. The activating receptors, FcγRI (CD64) and III (CD16), are expressed in both humans and mice, whereas FcγRIIa and IV are expressed in humans and mice, respectively. Activating FcγR contain cytosolic immunoreceptor tyrosine-based activation motifs that recruit kinases and phospholipases to promote inflammatory responses. The inhibitory receptor, FcγRIIb (CD32), is expressed on B cells and phagocytes. FcγRIIb has a cytosolic immunoreceptor tyrosine-based inhibition motif that recruits phosphatases for dampening of responses. It has been shown that, as IC levels rise, with the associated increase in activating FcγR occupancy, macrophages shift from a pro- to anti-inflammatory phenotype, as evidenced by reduced IL-12 and increased IL-10 production [69].

Zhu et al. showed that deficiency of CD16 in the hyperlipidemic apoE-knockout mouse model showed attenuated atherosclerotic lesions, reduced foam cell formation, without affecting the expression of other scavenger receptors. It was demonstrated that CD16 recognized MDA epitopes in MDA-LDL and CD16-MDA-LDL interaction resulted in induction of pro-inflammatory cytokine and chemokines [70].

Asciutto et al. reported recently that low levels of IgG autoantibodies against the native or MDA-modified apoB peptide p210 are associated with an increased risk of cardiovascular death in patients undergoing carotid endarterectomy [71]. The activation of macrophages by IgG-IC is determined by the balance between the triggering of activating ITAM-bearing FcγRs and the triggering of inhibitory ITIM-bearing FcγRIIB. The antigen size, concentration and IgG valence in the IC could be additional factors that influence macrophage activation [72].

OxLDL is poorly defined and neo-eptiopes are continually formed and degraded during the oxidation process so the density shift upon binding of β2GPI to in vitro oxLDL is unpredictable. However, we cannot exclude the presence of β2GPI in the investigated small LDL-IgG-IC. Theoretically, the density shift observed for one IgG molecule would be achieved upon binding of 3 β2GPI molecules per LDL particle. As the binding of β2GPI depends on negative charges of the binding partner LDL may appear as multiple target. Binding of IgG would shift the particle into small dense fraction or even out of LDL density range. However, we calculated that one single IgG would transport LDL particles from the dominant fraction exactly to the position where small LDL-IgG-IC have been detected. The presence of additional proteins in small LDL-IgG-IC would markedly affect its size and result in a more diffuse distribution due to compositional heterogeneity.

Bancells et al. analysed the proteome of electropositive LDL and electronegative LDL in blood of healthy subjects. LC-ESI MS/MS analysis of both LDL fractions identified up to 28 different proteins. Immunoglobulin lambda chain was detected in electronegative LDL which showed a higher content of most minor proteins [73].

Calculations based upon older data regarding the distribution of β2GPI among density fractions revealed that ~5% of total LDL is bound with one molecule of β2GPI or ~1% is bound by 5 molecule of β2GPI. In the studied healthy subjects ~2% of total β2GPI was found associated with the LDL fraction [74]. In contrast, it has been reported that in healthy controls no β2GPI is associated with LDL [75].

Several studies have shown that IgG anti-oxLDL/β2GPI antibody levels are higher in SLE patients than in healthy controls, and even higher in SLE patients with APS as compared to the ones without APS. Their correlation with atherosclerotic disease in patients with autoimmune diseases is still under investigation. The appearance of antibodies to β2GPI is associated with both SLE and APS. Circulating oxLDL/β2GPI IC and corresponding antibodies are found in sera of patients. OxLDL is an important factor in the acceleration of atherosclerosis in SLE and APS patients. The reason that it may be an accelerating factor is that while oxLDL is quickly removed from the circulation, oxLDL/β2GPI IC that form in these diseases due to the increased production of the antibodies persist for prolonged period of time, significantly increasing macrophage activation and foam cell formation. Immunostaining showed co-localization of oxLDL and β2GPI in atherosclerotic lesions supporting the notion that these complexes are deposited in the lesions and are atherogenic [76].

Considering this overall context the physiological and/or pathophysiological role (atherogenicity) of small LDL-IgG-IC appears unpredictable since their plasma residence time and susceptibility to undergo further modifications are presently unknown and may depend on various interacting factors.

## Conclusions

Our results show that the analysed small LDL-IgG-IC consist of one single IgG molecule per LDL particle and are not precipitable with PEG. The levels of small LDL-IgG-IC are low if compared to PEG precipitated IC. A prevalence of more dense LDL particles to form LDL-IgG-IC was not observed. IgG-IC of VLDL, IDL and Lp(a) were also detectable in lipoprotein subfractions. The primary autoimmunogenic epitope of small LDL-IgG-IC may be related to oxidative modification of apoB similar to that seen in human atherosclerotic plaques. Simvastatin reduced small LDL-IgG-IC levels more effectively than LDL-cholesterol and LDL-apoB levels and may therefore display therapeutic qualities beyond the lipid-lowering effect.

## Supporting Information

S1 Fig DELFIA setup and analysis. DELFIA setup to determine LDL-IgG-IC (**A**). DELFIA setup to determine apoB (**B**). Linearity and imprecision profile of the LDL-IgG-IC DELFIA assay. The assay system shows good linearity over a range from 0.1–50 μg/mL (based on total protein of LDL). The diagram displays sample counts per second (cps) vs. coefficient of variation (CV) (**C**).
(TIF)

S2 Fig Iodixanol gradient ultracentrifugation of human plasma and free proteins. Purified human IgG (**A**), human plasma (**B**) and an HRP-conjugated antibody (**C**) were fractionated following ultracentrifugation. The isolated fractions were transferred to nitrocellulose (**A**) or microtiter plates (**B** and **C**). Human IgG was detected by a specific antibody (**A** and **B**) and HRP-activity was measured directly (**C**). The fractionation step size of the displayed fractions was 3.0 mm. The results indicate that free (unbound) IgG molecules are detectable in subfractions ≥ #16. This observation ensures that the LDL-subfractions that generally contain the fraction of small LDL-IgG-IC are free from unbound human IgG.
(TIF)

S3 Fig Agarose electrophoresis of lipoprotein fractions isolated after iodixanol gradient ultracentrifugation and immunodetection of human IgG. A representative distribution pattern of human IgG among isolated subfractions (Std: control LDL-IgG-IC fraction) is shown (**A**). Electrophoresis of LDL-IgG-IC and free (unbound) human IgG (**B**).
(TIF)

S4 Fig Detection of oxidatively modified LDL with the monoclonal antibody OB/04 (directed against oxidation-specific epitopes). Affinity purified small LDL-IgG-IC (bound) and residual LDL (unbound; small LDL-IgG-IC depleted) were prepared from an LDL-subfraction rich in small LDL-IgG-IC. DELFIA counts of apoB levels were determined (setup shown in S1B Fig; sample dilution: 1:5.000) in the unbound and bound fraction. Levels of oxidation-related epitopes (same fractions) were determined by a direct DELFIA setup (microtitration plates coated with 100 μL of diluted fractions (1:50, 1:150 and 1:200)). Fluorescence counts (1 μg OB/04 antibody per well) were recorded after incubation with europium labelled goat anti-mouse IgG (#M-8770, Sigma Immunochemicals, St. Louis; USA). OB/04 DELFIA counts divided by the corresponding fluorescence counts of the apoB represent the concentration of modified apoB normalized to the concentration of apoB. The lower value has been transformed to 1.0 (unbound fraction). The level of oxidatively modified LDL was ~70-fold higher in small LDL-IgG-IC (normalized to apoB levels) if compared to the unbound fraction.
(TIF)

S5 Fig Distribution of small LDL-IgG-IC in LDL-subfractions isolated from 11 CAD patients. LDL (density: 1.019–1.065 g/mL) isolated from 6 mL plasma by preparative salt gradient ultracentrifugation was subsequently fractionated into six subfractions. DELFIA counts (representing LDL-IgG-IC) were converted into a percent value. For each subject and condition (pre-statin and post-statin) the fluorescence counts obtained for LDL 1–6 were summed and set to 100%. The distribution of small LDL-IgG-IC is presented as boxplot including all patients (n = 11) pre- and post-statin treatment. Boxplot displays minimum, lower quartile, median, upper quartile and maximum. ANOVA revealed significant differences (*p < 0.05; **p < 0.01; ***p < 0.001 (respective LDL-subfraction vs. LDL-subfraction #6); §§p < 0.01 (respective LDL-subfraction vs. LDL-subfraction #5)) (**A**). **Immunodetection of LDL-IgG-IC.** Representative blots (prepared from 1% agarose gels) from the same CAD patients illustrate that the major concentrations of small LDL-IgG-IC are located in the more dense LDL subfractions (LDL4—LDL6) (**B**). **Total amounts and particle ratio of small LDL-IgG-IC.** Values represent the total amounts of small LDL-IgG-IC (sum of total amounts of small LDL-IgG-IC measured in LDL-subfractions 1–6) expressed as apoB mass (μg small LDL-IgG-IC-apoB per mL of LDL fraction) in evaluable patients (n = 8) prior and after statin treatment (**C**). ApoB mass and particle ratio (IC-apoB as percentage of total apoB) of small LDL-IgG-IC were calculated based on DELFIA data (assumption: apoB affinity of the apoB-100 antibody is similar in small LDL-IgG-IC and native LDL). An averaged apoB DELFIA calibration curve was used to convert fluorescence counts from the LDL-IgG-IC DELFIA into apoB mass. Detection antibodies used in the DELFIA procedures applied for small LDL-IgG-IC and apoB determination are identical (see: S1A and S1B Fig).
(TIF)
