# Supplementary figures and images for: Simvastatin Efficiently Lowers Small LDL-IgG Immune Complex Levels: A Therapeutic Quality beyond the Lipid-Lowering Effect
Source: PLoS One. 2016 Feb 3;11(2):e0148210. doi: 10.1371/journal.pone.0148210 (PMC4739583; doi:10.1371/journal.pone.0148210)

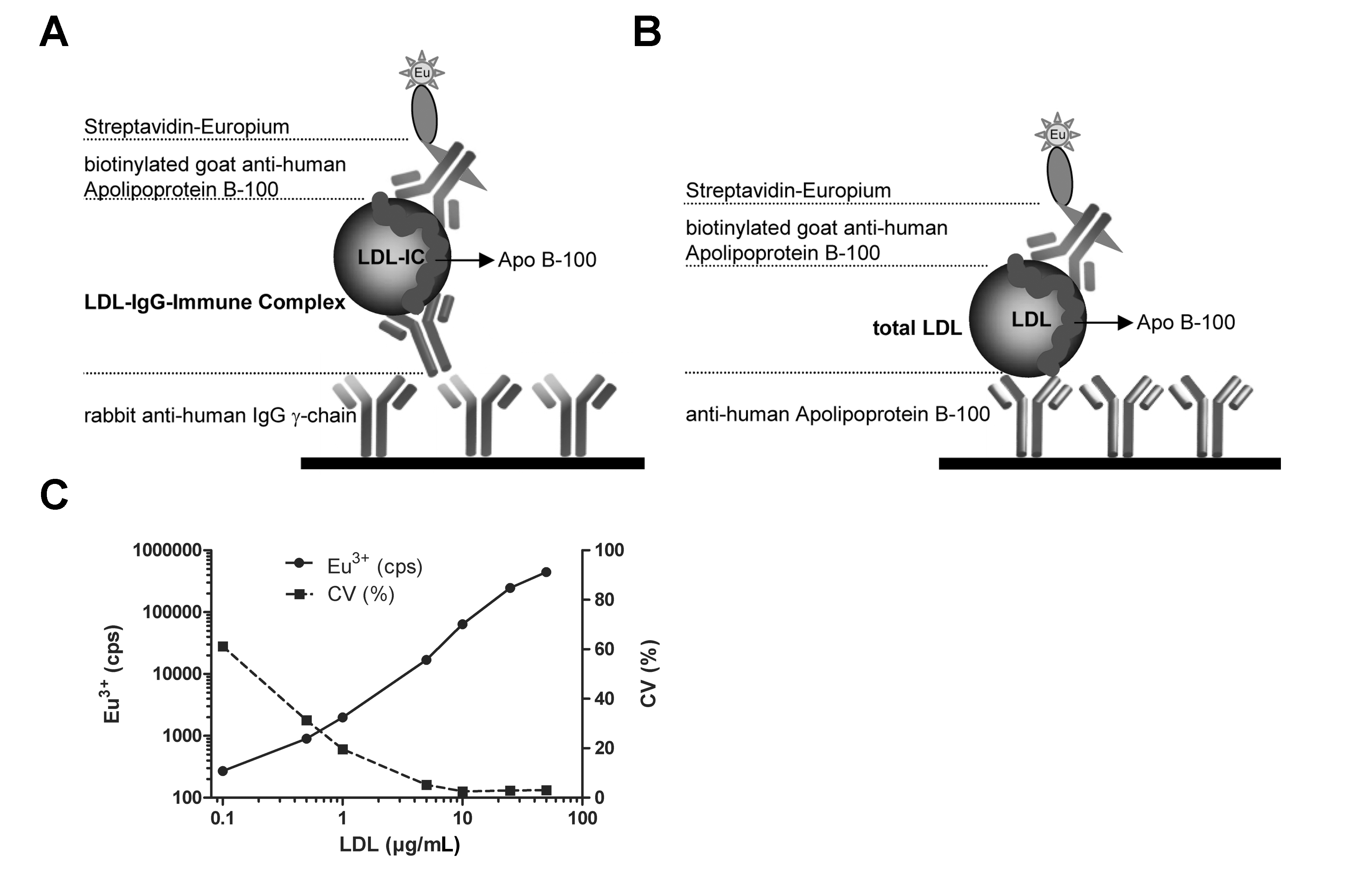

Supplement: S1 Fig — DELFIA setup to determine LDL-IgG-IC (A). DELFIA setup to determine apoB (B). Linearity and imprecision profile of the LDL-IgG-IC DELFIA assay. The assay system shows good linearity over a range from 0.1–50 μg/mL (based on total protein of LDL). The diagram displays sample counts per second (cps) vs. coefficient of variation (CV) (C). (TIF) [file pone.0148210.s001.tif]

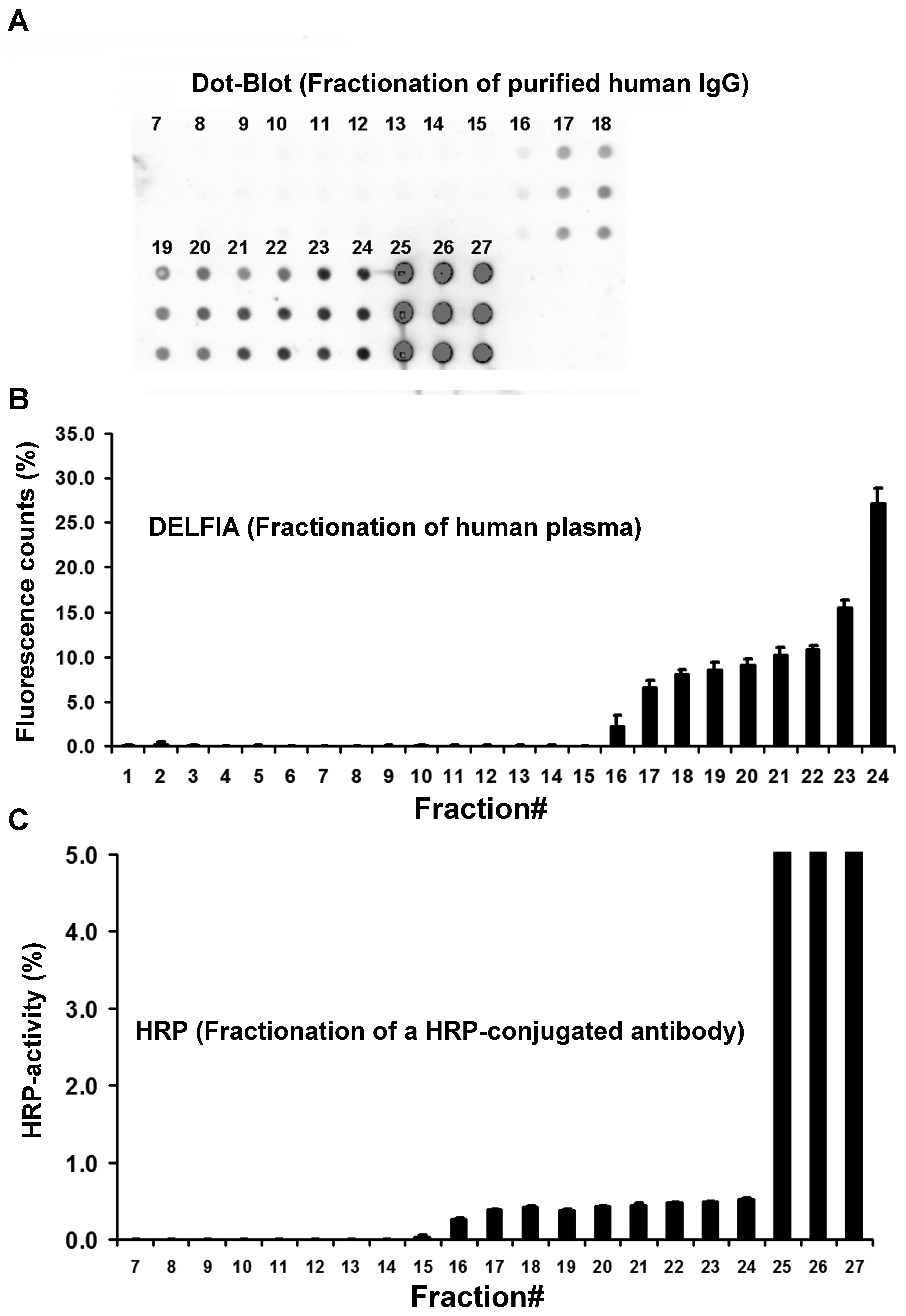

Supplement: S2 Fig — Purified human IgG (A), human plasma (B) and an HRP-conjugated antibody (C) were fractionated following ultracentrifugation. The isolated fractions were transferred to nitrocellulose (A) or microtiter plates (B and C). Human IgG was detected by a specific antibody (A and B) and HRP-activity was measured directly (C). The fractionation step size of the displayed fractions was 3.0 mm. The results indicate that free (unbound) IgG molecules are detectable in subfractions ≥ #16. This observation ensures that the LDL-subfractions that generally contain the fraction of small LDL-IgG-IC are free from unbound human IgG. (TIF) [file pone.0148210.s002.tif]

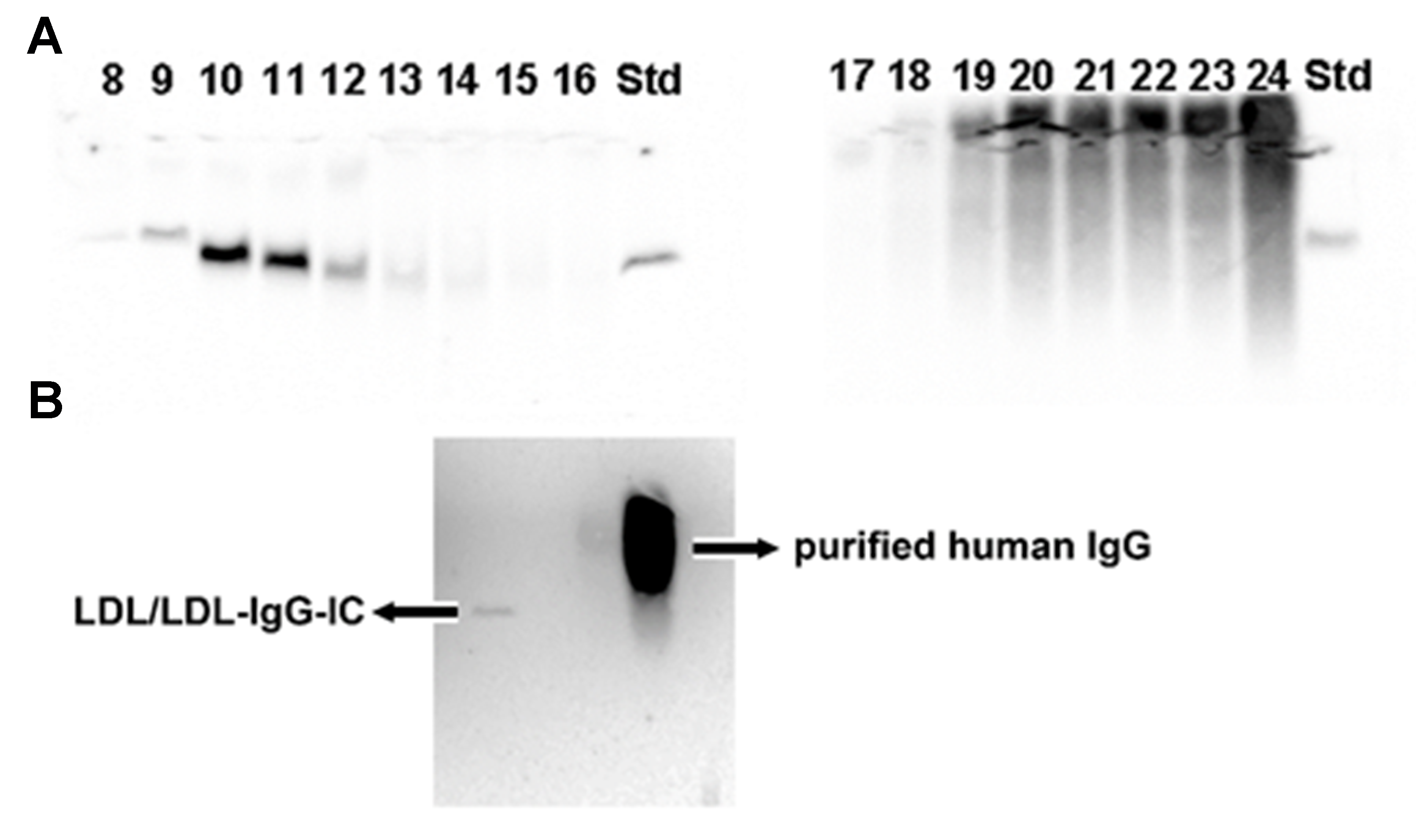

Supplement: S3 Fig — A representative distribution pattern of human IgG among isolated subfractions (Std: control LDL-IgG-IC fraction) is shown (A). Electrophoresis of LDL-IgG-IC and free (unbound) human IgG (B). (TIF) [file pone.0148210.s003.tif]

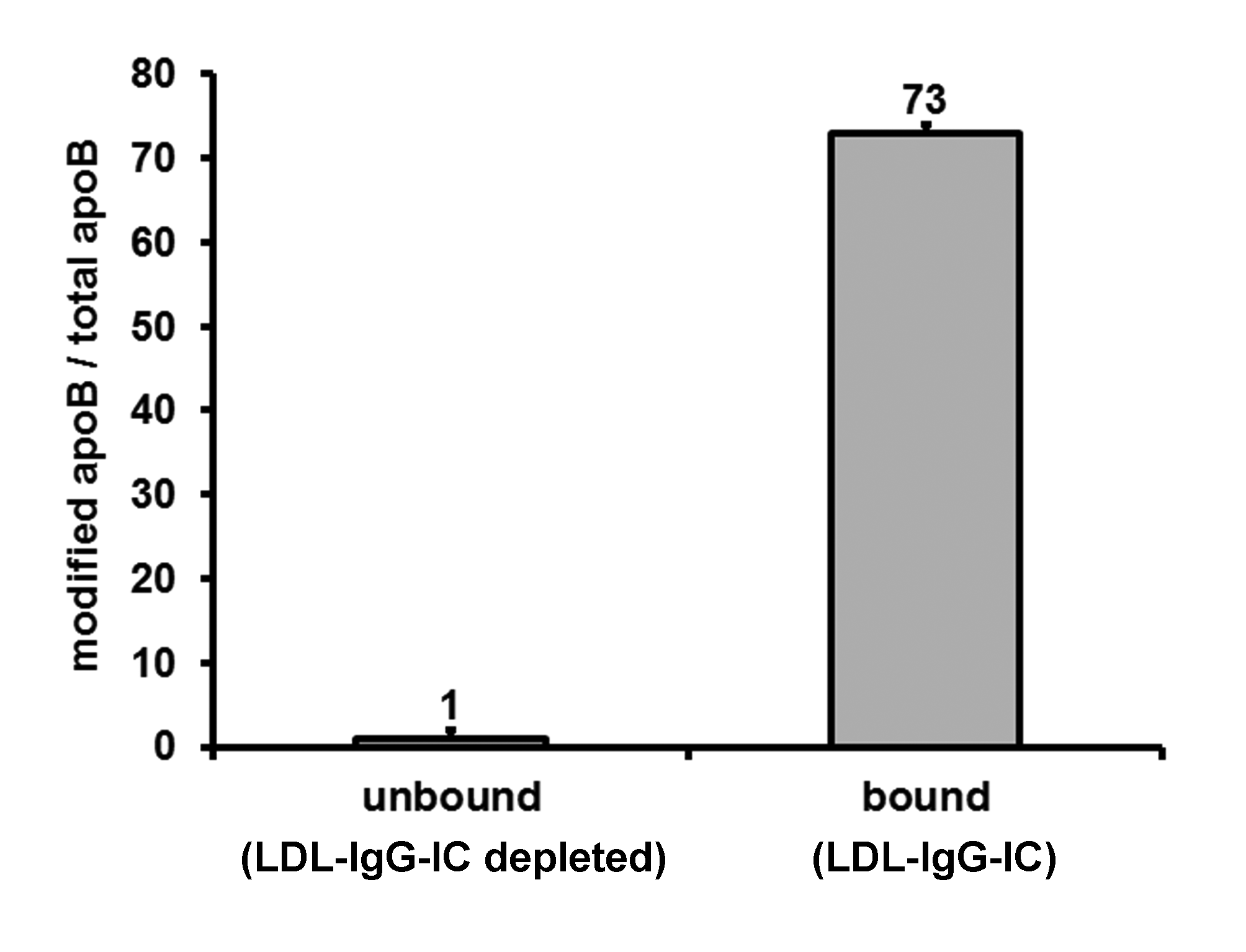

Supplement: S4 Fig — Affinity purified small LDL-IgG-IC (bound) and residual LDL (unbound; small LDL-IgG-IC depleted) were prepared from an LDL-subfraction rich in small LDL-IgG-IC. DELFIA counts of apoB levels were determined (setup shown in S1B Fig; sample dilution: 1:5.000) in the unbound and bound fraction. Levels of oxidation-related epitopes (same fractions) were determined by a direct DELFIA setup (microtitration plates coated with 100 μL of diluted fractions (1:50, 1:150 and 1:200)). Fluorescence counts (1 μg OB/04 antibody per well) were recorded after incubation with europium labelled goat anti-mouse IgG (#M-8770, Sigma Immunochemicals, St. Louis; USA). OB/04 DELFIA counts divided by the corresponding fluorescence counts of the apoB represent the concentration of modified apoB normalized to the concentration of apoB. The lower value has been transformed to 1.0 (unbound fraction). The level of oxidatively modified LDL was ~70-fold higher in small LDL-IgG-IC (normalized to apoB levels) if compared to the unbound fraction. (TIF) [file pone.0148210.s004.tif]

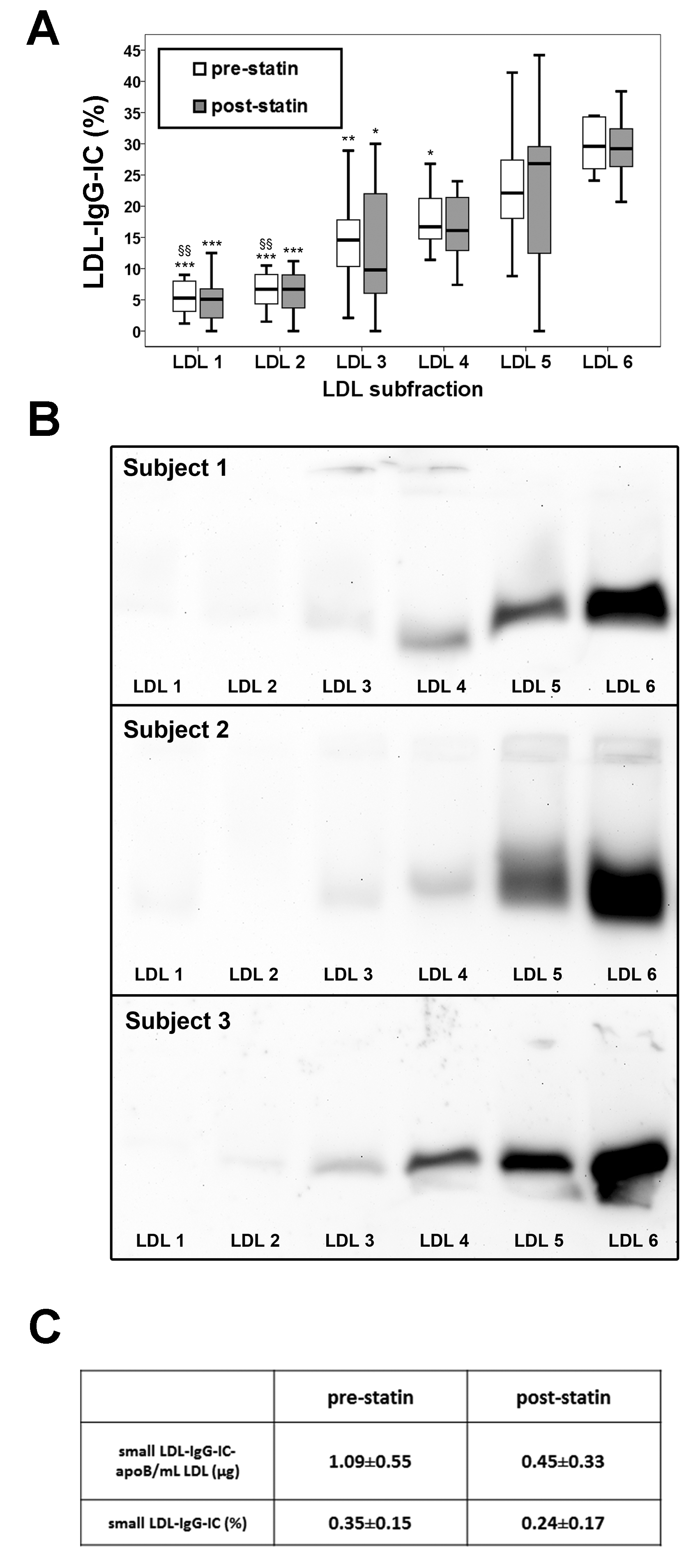

Supplement: S5 Fig — LDL (density: 1.019–1.065 g/mL) isolated from 6 mL plasma by preparative salt gradient ultracentrifugation was subsequently fractionated into six subfractions. DELFIA counts (representing LDL-IgG-IC) were converted into a percent value. For each subject and condition (pre-statin and post-statin) the fluorescence counts obtained for LDL 1–6 were summed and set to 100%. The distribution of small LDL-IgG-IC is presented as boxplot including all patients (n = 11) pre- and post-statin treatment. Boxplot displays minimum, lower quartile, median, upper quartile and maximum. ANOVA revealed significant differences (*p < 0.05; **p < 0.01; ***p < 0.001 (respective LDL-subfraction vs. LDL-subfraction #6); §§p < 0.01 (respective LDL-subfraction vs. LDL-subfraction #5)) (A). Immunodetection of LDL-IgG-IC. Representative blots (prepared from 1% agarose gels) from the same CAD patients illustrate that the major concentrations of small LDL-IgG-IC are located in the more dense LDL subfractions (LDL4—LDL6) (B). Total amounts and particle ratio of small LDL-IgG-IC. Values represent the total amounts of small LDL-IgG-IC (sum of total amounts of small LDL-IgG-IC measured in LDL-subfractions 1–6) expressed as apoB mass (μg small LDL-IgG-IC-apoB per mL of LDL fraction) in evaluable patients (n = 8) prior and after statin treatment (C). ApoB mass and particle ratio (IC-apoB as percentage of total apoB) of small LDL-IgG-IC were calculated based on DELFIA data (assumption: apoB affinity of the apoB-100 antibody is similar in small LDL-IgG-IC and native LDL). An averaged apoB DELFIA calibration curve was used to convert fluorescence counts from the LDL-IgG-IC DELFIA into apoB mass. Detection antibodies used in the DELFIA procedures applied for small LDL-IgG-IC and apoB determination are identical (see: S1A and S1B Fig). (TIF) [file pone.0148210.s005.tif]
